# Structured reporting of chest CT provides high sensitivity and specificity for early diagnosis of COVID-19 in a clinical routine setting

**DOI:** 10.1259/bjr.20200574

**Published:** 2020-11-27

**Authors:** Alexander Gross, Georg Heine, Martin Schwarz, Dorina Thiemig, Sven Gläser, Thomas Albrecht

**Affiliations:** 1 Department of Radiology and Interventional Therapy, Vivantes Klinikum Neukölln, Berlin, Germany; 2 Department of Pulmonary Medicine and Infectious Diseases, Vivantes Klinikum Neukölln, Berlin, Germany

## Abstract

**Objectives:**

Although chest CT has been widely used in patients with COVID-19, its role for early diagnosis of COVID-19 is unclear. We report the diagnostic performance of chest CT using structured reporting in a routine clinical setting during the early phase of the epidemic in Germany.

**Methods:**

Patients with clinical suspicion of COVID-19 and moderate-to-severe symptoms were included in this retrospective study. CTs were performed and reported before RT-PCR results (reference standard) became available. A structured reporting system was used that concluded in a recently described five-grade score (“CO-RADS”), indicating the level of suspicion for pulmonary involvement of COVID-19 from 1 = very low to 5 = very high. Structured reporting was performed by three Radiologists in consensus.

**Results:**

In 96 consecutive patients (50 male, mean age 64), RT-PCR was positive in 20 (21%) cases. CT features significantly more common in RT-PCR-positive patients were ground-glass opacities as dominant feature, crazy paving, hazy margins of opacities, and multifocal bilateral distribution (*p* < 0.05). Using a cut-off point between CO-RADS 3 and 4, sensitivity was 90%, specificity 91%, positive predictive value 72%, negative predictive value 97%, and accuracy 91%. ROC analysis showed an AUC of 0.938.

**Conclusions:**

Structured reporting of chest CT with a five-grade scale provided accurate diagnosis of COVID-19. Its use was feasible and helpful in clinical routine.

**Advances in knowledge:**

Chest CT with structured reporting may be a provisional diagnostic alternative to RT-PCR testing for early diagnosis of COVID-19, especially when RT-PCR results are delayed or test capacities are limited.

## Introduction

In the event of an infectious disease outbreak, quick and reliable early diagnosis is key for individual patient treatment as well as for disease containment, efficient employment of potentially limited healthcare resources, and protection of health-care workers.^1^ For COVID-19, a pandemic caused by Severe Acute Respiratory Syndrome Corona Virus 2 (SARS-CoV-2), reverse transcription–polymerase chain reaction (RT-PCR) from throat swabs, sputum, or bronchoalveolar lavage represents the diagnostic reference standard.^2–4^ Its exact sensitivity and specificity in a clinical setting, however, are not known as they depend not only on the test performance itself (which may vary between different sites), but also on viral load, sample quality and handling and timing of sample acquisition.^5–8^ Furthermore, RT-PCR tests may take up to several days and are not equally available in all parts of the world; test capacities may be limited even in highly developed countries.

Chest CT is a readily available diagnostic tool that has been widely used in patients with COVID-19. Various publications have shown characteristic CT features of COVID-19 pneumonia, multifocal patchy or polycyclic ground-glass opacities (GGO) and/or consolidations being the most frequent changes.^9–11^ Early reports from Europe have confirmed these observations.^12,13^ Chest CT changes may precede positive RT-PCR by several days in patients with initially false-negative RT-PCR results,^14–17^ and occur in up to 54% of asymptomatic patients.^18^ On the other hand, CT may be negative in some RT-PCR positive patients, especially at the early stage of the disease.^6,17,19^ Moreover, despite showing high sensitivities of up to 97%, early reports on specificity of CT for COVID-19 have been relatively disappointing, ranging from 25 to 56%.^6,20,21^ Data from Western countries on performance of chest CT in diagnosing COVID-19 are limited.^20,22,23^ While chest CT was recommended as major evidence for clinical diagnosis of COVID-19 in China, Western radiological societies including the American College of Radiology and the British Society of Thoracic Imaging advised against its regular use in initial patient workup.^24–27^The Fleischner Society, however, recently advocated the use of chest CT in patients with worsening respiratory status or in situations with limited RT-PCR testing capacities.^28^

Structured reporting including probability scales is increasingly used to objectify and standardise radiological reports in many areas.^29,30^ In an attempt to optimise the performance of chest CT, we introduced a structured reporting system in our hospital at an early phase of the epidemic, including a five-point probability scale (“CO-RADS”) for presence of COVID-19 pneumonia as proposed and recently published by the Dutch Radiological Society.^23,31^ In this retrospective single centre study, we analysed the clinical application of the CO-RADS for early diagnosis of COVID-19 in a large public hospital in Germany.

## Methods and materials

### Study population and inclusion criteria

We included consecutive patients with suspicion of COVID-19 with moderate-to-severe symptoms (body temperature >38°C, acute respiratory symptoms like cough or dyspnoea, respiratory rate >20/ min, peripheral capillary oxygen saturation <95%) undergoing chest CT in our hospital between March 22 and April 7, 2020. Patients presented either via the Emergency Department or were admitted to our hospital for unrelated conditions and developed symptoms suggestive of COVID-19 (as above) during their hospital stay. All patients were isolated and had a throat swab for RT-PCR and a chest CT shortly after admission, or when the clinical suspicion of COVID-19 was raised. Unstable patients requiring urgent invasive ventilation, who had chest CT later during their hospital stay, were not included. Moreover, patients with RT-PCR results available prior to CT or patients with COVID-19 pneumonia detected coincidentally on CT performed for other indications were not considered. A patient flow chart is given in Supplementary Material 1.

Supplementary Material 1.

Key clinical symptoms, respiratory parameters and laboratory findings were extracted from our digital hospital information system for all cases.

Waiver for approval and patient consent for this retrospective study were given by the local ethics committee.

### CT technique

Standard low-dose unenhanced or contrast-enhanced chest CT was performed using a 128-slice multidetector CT (iCT 128, Philips, Eindhoven) at 100 kVp (contrast-enhanced) or 120 kVp (unenhanced) and a dose right index of 8 with dose modulation. Contrast-enhanced chest CT was requested mainly when pulmonary embolism was considered a relevant differential diagnosis. It was performed after weight-adapted i.v. injection of 64–100 ml of contrast material (Iomeprol 400, Bracco, Italy). All images were iteratively reconstructed at axial 1 mm sections using a high-resolution kernel (lung window) and at axial 2 and 4 mm sections using a soft tissue kernel. Coronal and sagittal reformations were also obtained.

### Structured reporting and CO-RADS score

All CTs were reported in clinical routine shortly after CT images were obtained and before RT-PCR results were available by at least three board certified Radiologists by consensus, using a dedicated structured reporting system. This was put into place on March 22, 2020 based on our initial experiences with CT of COVID-19 pneumonia and on literature reports. The following CT-findings were systematically recorded: the presence of GGO, consolidation, crazy paving, reticulation and reverse halo; it was noted if GGO or consolidation was the dominant feature. The morphology of the single GGO and consolidations was characterized as hazy *vs* sharply delineated and as round or polycyclic *vs* irregular. Furthermore, the number of lesions as well as location and distribution of opacities were recorded: (1) peripheral (*i.e.* close to visceral pleural surfaces including the fissures) *vs* central (*i.e.* peribronchovascular); (2) upper *vs* lower lobe predominance; (3) ventral *vs* dorsal lung zone predominance; and (4) if lesions occurred bilaterally. Pleural effusions or lymphadenopathy (lymph nodes with a short axis diameter >1 cm) were noted as well. A complete list of CT findings assessed by the structured reporting template is shown in Supplementary Material 2.

Supplementary Material 2.

The structured reports concluded in a five-point scale indicating the probability of COVID-19 pneumonia. We found our self-developed five-grade scale structured report to be very similar to the CO-RADS system proposed by the Dutch Radiological Society online, thus we adapted ours to the official CO-RADS recently published by Prokop and colleagues.^23,31^ In brief, it is defined as follows: CO-RADS 1 = very low level of suspicion for pulmonary involvement by COVID-19; CO-RADS 2 = low level of suspicion for pulmonary involvement by COVID-19; CO-RADS 3 = equivocal findings for pulmonary involvement of COVID-19; CO-RADS 4 = high level of suspicion for pulmonary involvement by COVID-19; CO-RADS 5 = very high level of suspicion for pulmonary involvement by COVID-19.

In keeping with previous reports, ill-defined multifocal bilateral ground-glass opacities with or without consolidations, predominantly in peripheral or in both peripheral and central lung regions were considered typical of COVID-19.^9–11,23^ Crazy paving and reverse halo were regarded as rare, optional, but highly specific additional typical features. Pleural effusions or lymphadenopathy were considered atypical.

As described previously, CO-RADS 1 was assigned to normal CT and CT findings specific for an alternative non-infectious diagnosis (*e.g.* emphysema, tumour), stable to previous imaging (if performed and available) and without underlying obscuring lung pathology. CO-RADS 2 was given for lung alterations typical of infectious aetiology which are considered not consistent with COVID-19 (*e.g.* lobar pneumonia). CO-RADS 3 (equivocal findings) was chosen if (new or increased) features found in COVID-19 as well as in other viral pneumonias or non-infectious aetiologies were present (*e.g.* perihilar ground-glass without pleural effusion in absence of other typical CT findings). Moreover, it was used if there was a possible overlap between COVID-19 and a (possibly pre-existing) alternative diagnosis. CO-RADS 4 was assigned to findings similar to CO-RADS 5 but unilateral, without contact to the visceral pleura or in predominantly peribronchovascular location. In addition, this category was employed if highly suggestive findings were superimposed on severe diffuse pre-existing pulmonary abnormalities. CO-RADS 5 was used for (new or increased) CT findings typical of COVID-19 (as described above), in the absence of (possibly obscuring) severe diffuse underlying lung disease.^23,31^

All other radiological findings such as signs of typical pneumonia or pulmonary embolism were also reported. Radiologists had access to the clinical information provided in the CT request and to all other data in the hospital information system, including laboratory results if available at the time. However, radiologists were asked to base their report on CT findings only.

Out of hours, CO-RADS scoring was done by telereporting from home via remote access to our PACS system. All radiologists were trained prior to the introduction of the standardised reporting system by several joint sessions of literature review. Furthermore, suspected cases were regularly shown and discussed in our morning round.

### Statistical analysis

Clinical and laboratory data as well as CT findings of RT-PCR positive and negative patients were compared using Mann-Whitney U-test (numerical data) or Fisher’s exact test (categorical data). CO-RADS score was compared with RT-PCR results, which served as the reference standard. CO-RADS score results were used for receiver operating characteristics (ROC) analysis. Sensitivity, specificity, positive predictive value (PPV), negative predictive value (NPV) and accuracy, including 95% CI, were calculated for the best cut-off point of CO-RADS score.

SPSS 19 (IBM Corp., Armonk, USA) was used for statistical analysis. A two-tailed *p*-value < 0.05 was considered statistically significant.

## Results

We included a total of 96 patients (50 males and 46 females, age 17–98 years, mean age 64 years) with suspicion of COVID-19. Of these, 90 patients were admitted as in-patients via the Emergency Department, and six were admitted for unrelated conditions and developed symptoms suggestive of COVID-19 during their hospital stay. RT-PCR was positive in 20 (21%) and negative in the remaining 76 patients (79%). In one patient with CO-RADS 5 and negative initial RT-PCR from throat swab, the highly suggestive CT changes prompted a second test from sputum which turned out positive. RT-PCR results were available after a mean of 19 h (maximum 5 days). Patient details are summarised in Table 1. Fever, normal leukocyte counts and increase in lactate dehydrogenase were significantly more common in SARS-CoV-2 positive patients.

**Table 1.** Patient details

		RT-PCR positive	RT-PCR negative	
		***n* = 20**	***n* = 76**	*p* value
**Characteristics**				
Age, years		56.9 (20.5)	65.8 (18.9)	0.040
Sex	Male	14 (70%)	36 (47%)	0.083
	Female	6 (30%)	40 (53%)	
**Symptoms**				
Fever (>38°C)		17 (85%)	42 (55%)	0.020
Cough		9 (45%)	38 (50%)	0.803
Dyspnoea		7 (35%)	30 (39%)	0.800
Abdominal symptoms		4 (20%)	15 (20%)	0.979
**Respiratory parameters**				
Respiratory rate, /min		20 (6.7)	20 (6.2)	0.865
Peripheral capillary oxygen saturation, %		90 (11.5)	93 (6.1)	0.594
**Laboratory results**				
Leukocyte count, x 10^9^/L		6.69 (2.90)	13.50 (8.68)	0.001
Lymphocyte count, x 10^9^/L		0.99 (0.47)	1.21 (0.79)	0.454
Lactate dehydrogenase, U/L		383 (170)	298 (140)	0.029
Procalcitonin, µg/L		0.36 (0.53)	3.29 (6.92)	0.252
C-reactive protein, mg/L		89.6 (98.6)	89.2 (94.8)	0.962

Data are mean (standard deviation) or n (%). RT-PCR = reverse transcription-polymerase chain reaction.

In total, 58 unenhanced and 38 contrast-enhanced CTs were performed. CT findings of RT-PCR positive and negative patients are shown in Table 2. Lung opacities were found in 19 of the 20 SARS-CoV-2-positive and 56 of the 76 negative patients. The following features were significantly more frequent in COVID-19 patients: GGO as the dominant feature, crazy paving, hazy margins of opacities and multifocal (more than three lesions) as well as bilateral distribution (*p* < 0.05). Conversely, consolidation as the dominant feature, GGO as a non-dominant feature, sharp margins, smaller number of lesions (up to three), and unilateral distribution were more frequently seen in negative patients (*p* < 0.05). All other morphological features did not differ significantly between the two groups. Pleural effusions were present in 15% of RT-PCR positive and 32% of RT-PCR negative patients (*p* = 0.172) and lymphadenopathy in 30 and 21%, respectively, (*p* = 0.387).

**Table 2.** CT findings. Number of patients with certain findings (*e.g.,* ground-glass opacities – GGO) related to the number of patients with any lung opacities present. For the main morphological features of COVID-19 pneumonia, GGO and consolidation, it was distinguished which of the two features was the dominant one. (The other morphological entities – crazy paving, reticulations and reversed halo – were additional features and never dominant.) “Margin” and “configuration” refer to GGO or consolidations, depending on which feature was dominant

		RT-PCR positive	RT-PCR negative	
Lung opacities present		***n* = 19**	***n* = 56**	
No lung opacities present		***n* = 1**	***n* = 20**	
				*p* value
**Ground-glass opacity (GGO)**	Present (dominant or non-dominant feature)	18 (95%)	46 (82%)	0.271
	Dominant feature	14 (74%)	21 (38%)	0.008
	Non-dominant feature	4 (21%)	25 (45%)	0.020
**Consolidation**	Present (dominant or non-dominant feature)	15 (79%)	47 (84%)	0.727
	Dominant feature	5 (26%)	34 (61%)	0.016
	Non-dominant feature	10 (53%)	13 (23%)	0.078
**Crazy paving**		12 (63%)	1 (2%)	0.001
**Reticulations**		3 (16%)	10 (18%)	1.000
**Reversed halo**		0 (0%)	2 (4%)	1.000
**Number of lesions**	1	0 (0%)	9 (16%)	0.101
	2–3	0 (0%)	13 (23%)	0.030
	>3	19 (100%)	34 (61%)	0.001
**Hazy margin of GGO or consolidations**		16 (84%)	29 (52%)	0.015
**Round or polycyclic configuration of GGO or consolidations**		11 (58%)	18 (32%)	0.101
**Bilateral distribution**		19 (100%)	31 (55%)	0.001
**Distribution central *vs* peripheral**	Peripheral only	5 (26%)	19 (34%)	0.777
	Peripheral and central	14 (74%)	33 (59%)	0.286
	Central only	0 (0%)	4 (7%)	0.567
**Distribution upper *vs* lower lobe**	Upper lobe dominant	1 (5%)	13 (23%)	0.100
	Lower lobe dominant	10 (53%)	25 (45%)	0.602
	No upper/lower lobe dominance	8 (42%)	18 (32%)	0.578
**Distribution ventral/dorsal**	Ventral dominant	1 (5%)	3 (5%)	1.000
	Dorsal dominant	13 (68%)	34 (61%)	0.784
	No ventral/dorsal dominance	5 (26%)	17 (30%)	0.777

Data are number of patients (%, referred to number of patients with lung opacities). RT-PCR = reverse transcription-polymerase chain reaction.

**Figure 1.**
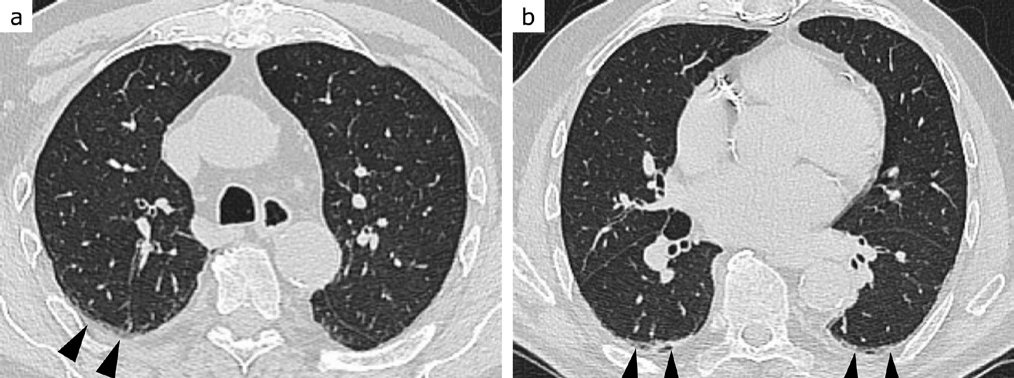
CO-RADS 1. 80-year-old patient with dyspnoea and chest pain, moderately elevated C-reactive protein and leucocytosis. Except for discreet bilateral dystelectasis (arrowheads, a and b), lungs were unremarkable. Acute coronary syndrome and urinary tract infection were diagnosed. RT-PCR for SARS-CoV-2 returned negative

**Figure 2.**
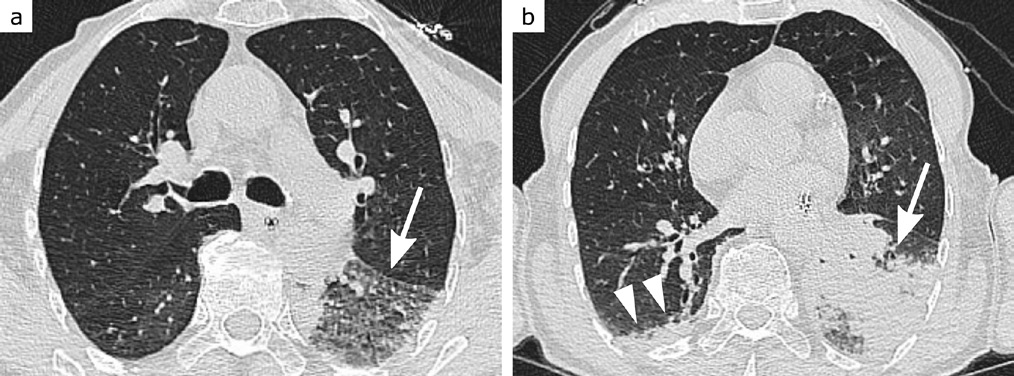
CO-RADS 2. 80-year-old patient with dementia, highly elevated C-reactive protein and slightly elevated leukocyte levels. Extensive ground-glass opacities and consolidation confined to the left lower lobe (arrows, a and b), leading to the diagnosis of typical lobar pneumonia. There is an additional small peripheral consolidation in the right lower lobe (arrowheads, b), leaving a low probability for COVID-19 pneumonia. RT-PCR for SARS-CoV-2 returned negative

**Figure 3.**
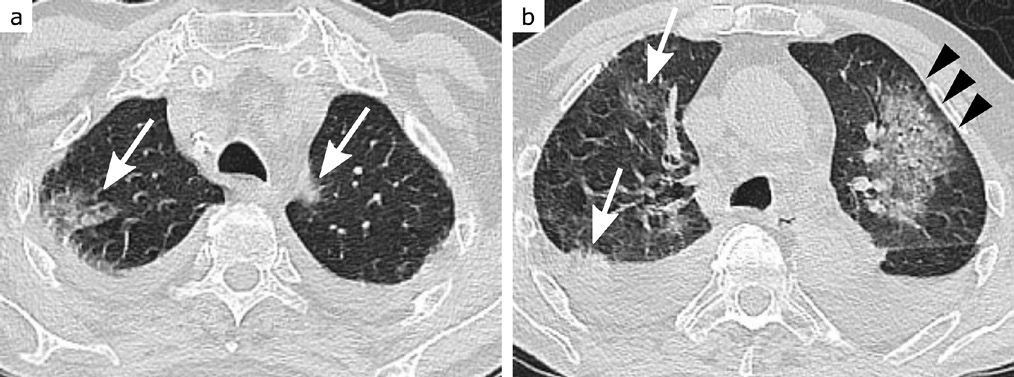
CO-RADS 3. 85-year-old patient with dyspnoea, moderately elevated C-reactive protein and normal leukocyte levels. Extensive ill-defined consolidation with surrounding ground-glass opacities (GGO) in the left upper lobe, sparing the subpleural space (arrowheads, b), raises the suspect of lobar pneumonia. Bilateral patchy both subpleural and central GGO (arrows, a and b), however, are in favour of COVID-19 pneumonia. Additional bilateral pleural effusions (probably due to pre-existing congestive heart failure) further complicate the situation. RT-PCR for SARS-CoV-2 returned negative

**Figure 4.**
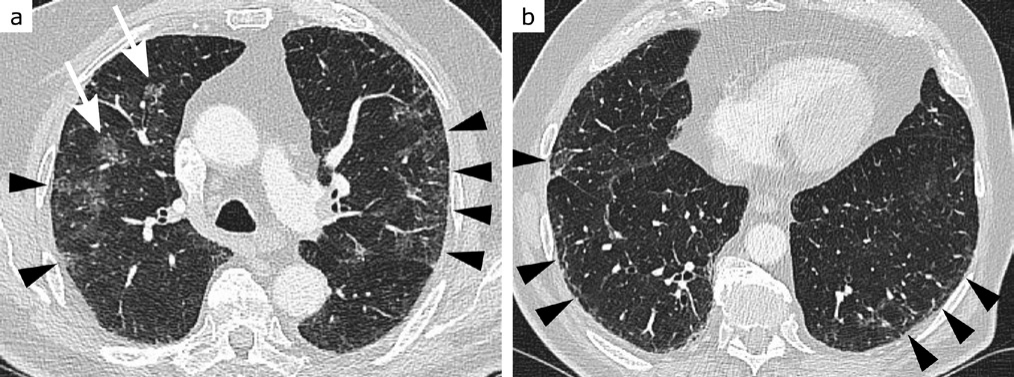
CO-RADS 4. 71-year-old patient with dyspnoea, highly elevated C-reactive protein level, normal leukocyte count and lymphopenia. Bilateral patchy ground-glass opacities (GGO) in peripheral (arrowheads, a and b) and central (arrows, a) location, typical for COVID-19 pneumonia. GGO appear very mild, however, especially in lower lung zones (b, here more appearing like reticulations), leaving some uncertainty. This is probably due to the moderate centrilobular emphysema. RT-PCR for SARS-CoV-2 returned positive

**Figure 5.**
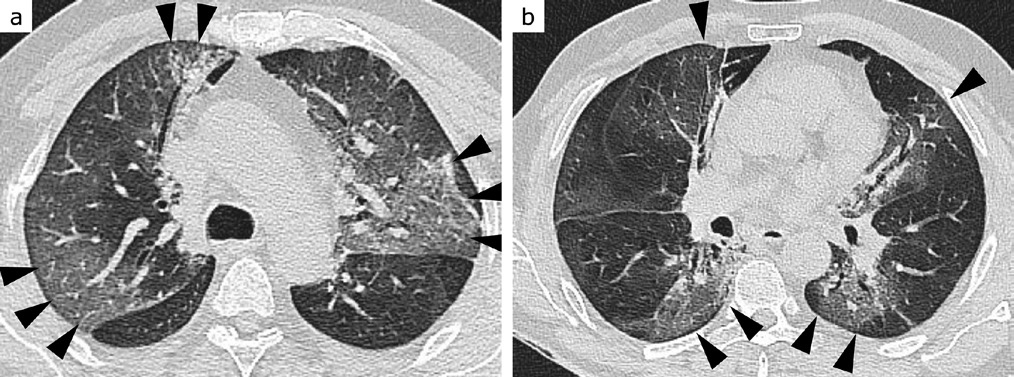
CO-RADS 5, ground-glass type. 54-year-old patient with headache, general weakness, fever and cough starting 3 days before the CT scan. Highly elevated C-reactive protein level, moderately elevated leukocyte count and lymphopenia. Geographic ground-glass opacities (arrowheads, a and b) extending from the subpleural space to central areas in both upper and lower lung zones, indicating COVID-19 pneumonia. RT-PCR for SARS-CoV-2 returned positive

**Figure 6.**
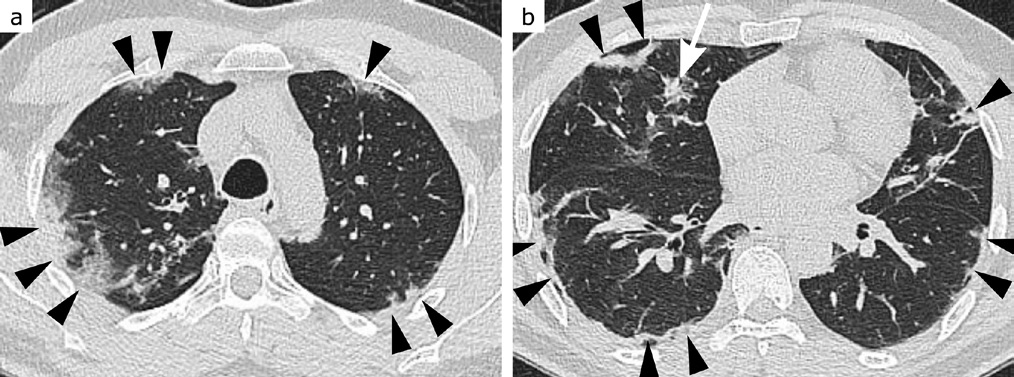
CO-RADS 5, consolidation type. 51-year-old patient with fever, cough and dyspnoea starting 8 days before the CT scan. Moderately elevated C-reactive protein and normal leukocyte levels. In upper lung zones (a) rounded, in lower lung zones (b) streaky consolidations in predominantly peripheral (arrowheads, a and b), but also central (arrow, b) location, indicating COVID-19 pneumonia. RT-PCR for SARS-CoV-2 returned positive

Of the 20 RT-PCR-positive patients, 18 (90%) had CO-RADS score 4 or 5, while two (10%) of them had CO-RADS score 1 or 2: one of these had a normal chest CT (CO-RADS 1), the other one had a severe scoliosis with related atelectasis of the right lower lobe and consolidation in the left upper lobe possibly obscuring other lung pathology (CO-RADS 2). Of the 76 RT-PCR negative patients, 62 (82%) had CO-RADS score 1 or 2 and seven (9%) were scored as 4 or 5. Of note is that all seven patients with CO-RADS score 3 (“indeterminate”) were RT-PCR-negative. The numbers of RT-PCR positive and negative patients per CO-RADS score are shown in Table 3. Examples of CT images for CO-RADS scores 1–5 are given in Figures 1–6.

**Table 3.** CO-RADS score

	RT-PCR positive	RT-PCR negative
	***n* = 20**	***n* = 76**
**CO-RADS 1: very low level of suspicion**	1 (5%)	43 (57%)
**CO-RADS 2: low level of suspicion**	1 (5%)	19 (25%)
**CO-RADS 3: equivocal findings**	0 (0%)	7 (9%)
**CO-RADS 4: high level of suspicion**	2 (10%)	6 (8%)
**CO-RADS 5: very high level of suspicion**	16 (80%)	1 (3%)

Data are number of patients (%). RT-PCR = reverse transcription-polymerase chain reaction.

**Figure 7.**
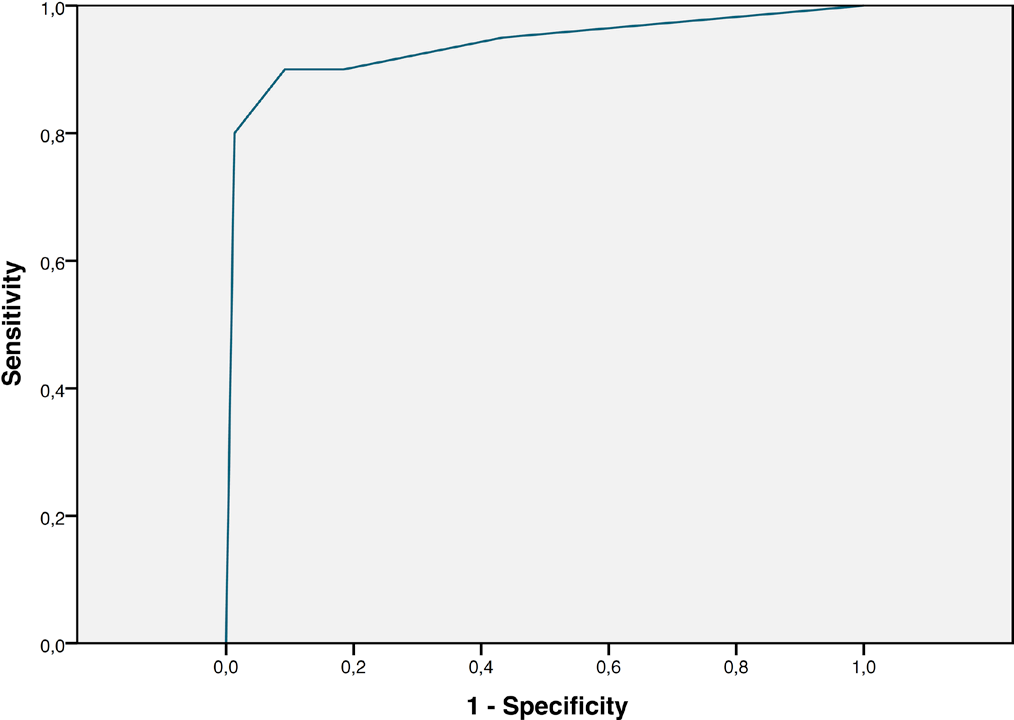
Receiver operating characteristics (ROC) analysis. ROC curve for detecting COVID-19 on CT scans using the CO-RADS score (five-point scale). RT-PCR-test results for SARS-CoV-2 served as reference standard. The area under the curve was 0.938.

Figure 7 shows the ROC curve for the CO-RADS of our study population. The area under the curve was 0.938. Using a lower cut-off point of CO-RADS 4, that is, when CO-RADS 4 and 5 were considered COVID-19, sensitivity was 90% (95% CI: 72–98%), specificity 91% (95% CI: 83–96%), PPV 72% (95% CI: 53–87%), NPV 97% (95% CI: 92–100%) and accuracy 91% (84–95%) using RT-PCR results as the reference. For comparison, using a lower cut-off point of CO-RADS 3, that is, when CO-RADS 3 (“indeterminate”) and higher were considered COVID-19, diagnostic performance was slightly lower: In this case, sensitivity was 90% (95% CI: 72–98%), specificity 82% (95% CI: 72–98%), PPV 56% (95% CI: 39–72%), NPV 97% (95% CI: 91–99%) and accuracy 83% (95% CI: 75–90%).

For patients with CO-RADS score 1–3 CT provided alternative diagnoses (Table 4). The most common were typical pneumonia, acute decompensated heart failure and pulmonary embolism. Contrast-enhanced CT was performed in 10 of the 20 SARS-CoV-2 positive patients. No pulmonary embolism was detected in any of these cases.

**Table 4.** CT diagnoses of SARS-CoV-2 negative patients. In some of these 69 patients, more than one alternative diagnosis was made by CT

	CO-RADS 1–3 (“true negatives”)
	***n* = 69**
**Thoracic CT diagnoses**	
Lobar/segmental pneumonia	22
Bronchopneumonia	13
Acute decompensated heart failure	9
Pulmonary embolism	4
Septic pulmonary emboli	1
Pleural empyema	1
Miliary tuberculosis	1
Neoplasia	3
Interstitial lung disease	1
Mucoid impaction with atelectasis	1
Contained rupture of aortic ulcer	1
Rib fractures	1
No acute pulmonary pathology	19
**Extrathoracic CT diagnoses**	
Acute pancreatitis	2
Pyelonephritis	2
Acute colonic diverticulitis	1

Data are number of patients.

## Discussion

In this study, we describe the use of a structured CT reporting system for early diagnosis of COVID-19 in clinical routine. Using a simple five-point scale (CO-RADS) to indicate the probability of COVID-19 pneumonia and RT-PCR as reference standard, chest CT had a high sensitivity of 90% and specificity of 91% in our retrospective analysis of 96 consecutive patients with clinical suspicion of COVID-19.

The diagnostic performance of chest CT with structured reporting in our study was very similar (AUC 0.938) to the results of the initial publication of CO-RADS, which reported an AUC of 0.91.^23^ While in the latter study CT scans were re-read and scored retrospectively by eight independent blinded readers, we applied CO-RADS in a clinical routine setting: the CO-RADS score was determined as part of the clinical report by at least three board certified radiologists in consensus shortly after CT was performed and before RT-PCR tests were available.

Most other previous publications on the diagnostic performance of CT for early diagnosis without structured reporting had similar sensitivity, but much lower specificity: in a large retrospective study from Wuhan, China, sensitivity was 97% and specificity 25% in 1014 patients with a proportion of RT-PCR-positive patients of 59%.^6^ In a similar retrospective analysis from China, sensitivity was 92% and specificity 53% in 103 patients with a prevalence of COVID-19 of 85%.^21^ In a prospective study from Italy, sensitivity was 97% and specificity 56% in a series of 158 consecutive patients admitted to the emergency department undergoing CT, with a prevalence of COVID-19 of 39%.^20^ Only one recent study showed a diagnostic performance on par with our results (sensitivity 87%, specificity 94%) in a retrospective series of 192 consecutive patients (43% SARS-CoV-2-positive) scored by two cardiothoracic radiologists without standardised reporting.^22^

We assume that the high specificity in our study is due to our structured reporting system. Structured reporting facilitates clear and concise interpretation and communication of imaging results, helps to establish diagnostic and therapeutic recommendations and ultimately improves patient care.^29,32,33^ Five-grade scales of probability are known from other established reporting classifications such as BI–RADS for breast cancer or PI–RADS for prostate cancer.^29,34,35^ Other, similar systems have been proposed for CTs evaluating the presence of COVID-19 pneumonia.^36–38^ Practicability, reliability and usefulness of such systems depend largely on how the assignment of patients to different categories is defined. Probability scales have the advantage that the reporting Radiologist expresses likelihood for the diagnosis instead of making a binary positive or negative diagnosis. Different cut-off points can then be used to find the threshold for best test performance. We found the best diagnostic results, if a cut-off point of CO-RADS 4 and higher was used, that is, when indeterminate cases were counted as negative. This implies that a relatively high threshold for CT findings should be used and patients should only be regarded as positive, if findings suggestive of COVID-19 outweigh those indicative of alternative diagnoses.

Although definitive diagnosis of COVID-19 is made by RT-PCR for SARS-CoV-2, CT plays an important role in diagnosing the disease in several ways as recently discussed in a multinational consensus statement from the Fleischner Society.^28^ According to our results, it can provide a swift provisional diagnosis or exclusion of COVID-19 in symptomatic patients requiring hospitalisation with high accuracy, while RT-PCR results may take several days (up to five days in our cohort). CT can be particularly useful in regions with limited availability of RT-PCR.^28^ In China, new diagnostic criteria that included CT findings were introduced by national guidelines at the height of the epidemic on 12 February 2020, to ensure timely isolation and treatment, because of the delays associated with RT-PCR-testing, which led to a surge of diagnoses of COVID-19.^24,39^ Although this recommendation was later reversed, it underlines the potential role for CT when laboratory capacities are stretched.

Special attention should be paid to patients with typical CT findings of COVID-19, but negative RT-PCR.^28^ In this situation RT-PCR should be repeated. There are several reports of patients with initially false negative RT-PCR, that became positive when (repeatedly) re-tested based on CT.^14–16^ Our series included one such case.

Early diagnosis is not only relevant for isolation of patients with COVID-19 but also has important therapeutic implications for patients without findings of COVID-19.^28^ This latter group was relatively large in our cohort (79% of patients) and included cases of typical, presumably bacterial pneumonia, acute decompensated heart failure, pulmonary embolism or tuberculosis, all requiring fast treatment. CT-enabled prompt diagnosis and therapy for these patients before RT-PCR results became available.

Recently, there have been a number of publications reporting high rates of venous thromboembolic complications in critically ill patients with severe COVID-19.^40,41^ In this study, contrast-enhanced CT was performed in 10 of the 20 SARS-CoV-2-positive patients. No pulmonary embolism was detected in any of these cases. Therefore, venous thromboembolic events do not seem to play a major role in the early phase of COVID-19.

Our study has limitations. It was retrospective in nature and had a limited number of patients, particularly in the COVID-19 group. On the other hand, our RT-PCR negative group was relatively large with 76 patients, suggesting good reliability with regards to the high specificity found. Despite our encouraging results, we regard the CO-RADS scoring system as provisional. It was introduced rapidly at the beginning of the epidemic in Germany in an attempt to cope with the expected surge of patients as adequately as possible, based on the scientific data available at the time and our own, albeit limited early experience.^6,9–11,19,31,42^ Publications on CT for COVID-19 are currently rapidly evolving, including proposals for structured reporting schemes as well as the use of artificial intelligence.^15,23,36,43^ More data and collaboration of involved centres will be required to reach an optimal, generally accepted and more extensively validated structured reporting system.

## Conclusion

In conclusion, the use of structured reporting with a five-grade scoring system (CO-RADS) allows chest CT to diagnose COVID-19 pneumonia with high sensitivity and specificity in a clinical routine setting. Although definitive diagnosis is made by RT-PCR, CT is able to provide a swift and relatively accurate provisional diagnosis or exclusion of COVID-19 in symptomatic patients requiring hospitalisation. This is all the more relevant in situations when RT-PCR results are delayed or RT-PCR testing capacities are limited. Moreover, it may detect patients with COVID-19 pneumonia and initially false-negative RT-PCR, and provide valuable diagnostic information for patients without COVID-19. Larger prospective trials will be required to optimise and validate standardised reporting systems.
