# Supplementary figures and images for: Structured reporting of chest CT provides high sensitivity and specificity for early diagnosis of COVID-19 in a clinical routine setting
Source: Br J Radiol. 2020 Nov 27;94(1117):20200574. doi: 10.1259/bjr.20200574 (PMC7774695; doi:10.1259/bjr.20200574)

## Supplementary Material 1: Patient flow chart

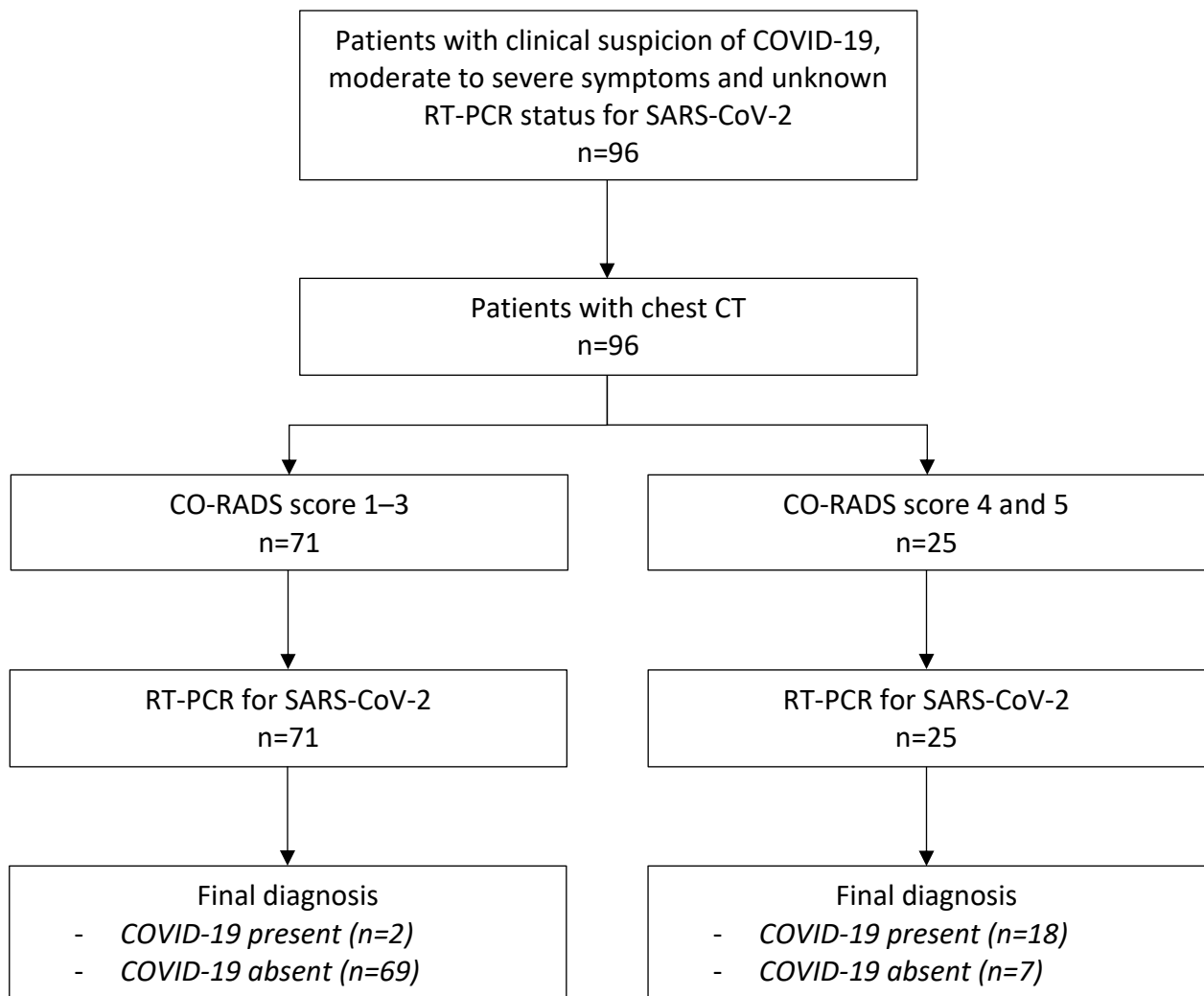

Supplement: Supplementary Material 1. [file bjr.20200574.suppl-01.pdf]
