# Supplementary material for: Structured reporting of chest CT provides high sensitivity and specificity for early diagnosis of COVID-19 in a clinical routine setting
Source: Br J Radiol. 2020 Nov 27;94(1117):20200574. doi: 10.1259/bjr.20200574 (PMC7774695; doi:10.1259/bjr.20200574)
Supplement: Supplementary Material 2. [file bjr.20200574.suppl-02.pdf]

Supplementary Material 2: COVID-19 CT structured reporting form

**Category**	**Options**
CT technique	Unenhanced
	Contrast-enhanced
Time course	First CT for COVID-19
	Follow-up CT for COVID-19
Lung structure	Unremarkable
	Mild emphysema
	Moderate emphysema
	Severe emphysema
	Lung fibrosis
Dominant lung opacity	Ground-glass opacity
	Consolidation
Non-dominant lung opacities	Ground-glass opacity
	Consolidation
	Crazy paving
	Reticulations without crazy paving
	Reversed halo
Number of lesions	1
	2–3
	>3
Configuration of ground-glass opacities / consolidations	Round or polycyclic
	Not round or polycyclic
Margin of ground-glass opacities / consolidations	Sharp
	Hazy
Distribution (unilateral/bilateral)	Left
	Right
	Bilateral
Distribution (central/peripheral)	Peripheral only
	Peripheral and central
	Central only
Distribution (upper/lower lobe)	Upper lobe dominant
	Lower lobe dominant
	No upper/lower lobe dominance
Distribution (ventral/dorsal)	Ventral dominant
	Dorsal dominant
	No ventral/dorsal dominance
Overall extent	Mild
	Moderate
	Severe
Other pulmonary findings	*Free text*

Pleural effusions	None
	Small (<1 cm)
	Moderate (1–3 cm)
	Extensive (>3 cm)
Side of pleural effusions	Left
	Right
	Bilateral
Enlarged lymph nodes	No
	Yes (>1 cm)
Sites of enlarged lymph nodes	Mediastinal
	Hilar (uni-/bilateral)
Cardiovascular structures	Unremarkable
	Central pulmonary embolism (left/right/bilateral)
	Peripheral pulmonary embolism (left/right/bilateral)
	Free text
Heart	Normal size
	Enlarged
Medical devices	*Free text*
CO-RADS score	CO-RADS 1: very low level of suspicion
	CO-RADS 2: low level of suspicion
	CO-RADS 3: equivocal findings
	CO-RADS 4: high level of suspicion
	CO-RADS 5: very high level of suspicion
Overall disease severity	Mild
	Moderate
	Severe
Additional diagnoses and comments	*Free text*
